# Saxitoxin Group Toxins Accumulation Induces Antioxidant Responses in Tissues of *Mytilus chilensis*, *Ameghinomya antiqua*, and *Concholepas concholepas* during a Bloom of *Alexandrium pacificum*

**DOI:** 10.3390/antiox11020392

**Published:** 2022-02-15

**Authors:** Javiera Oyaneder-Terrazas, Diego Figueroa, Oscar F. Araneda, Carlos García

**Affiliations:** 1 Laboratory of Marine Toxins, Physiology and Biophysics Program, Institute of Biomedical Sciences, Faculty of Medicine, Universidad de Chile, Santiago 8380000, Chile; javiera.oyaneder@usach.cl (J.O.-T.); d.figueroa@cienciavida.org (D.F.); 2 Integrative Laboratory of Biomechanics and Physiology of Effort, Kinesiology School, Faculty of Medicine, Universidad de Los Andes, Santiago 8320000, Chile; ofaraneda@miuandes.cl

**Keywords:** saxitoxin, oxidative stress, antioxidant defenses, bivalves, gastropods, *Alexandrium pacificum*

## Abstract

Saxitoxin (STX) group toxins consist of a set of analogues which are produced by harmful algal blooms (HABs). During a HAB, filter-feeding marine organisms accumulate the dinoflagellates and concentrate the toxins in the tissues. In this study, we analyze the changes in antioxidant enzymes and oxidative damage in the bivalves *Mytilus chilensis* and *Ameghinomya antiqua*, and the gastropod *Concholepas concholepas* during a bloom of *Alexandrium pacificum*. The results show that during the exponential phase of the bloom bivalves show an increase in toxicity and activity of antioxidant enzymes (superoxide dismutase, catalase, glutathione peroxidase, and glutathinoe reductase, *p* < 0.05), while in the gastropods, increased activity of antioxidant enzymes was associated with the bioaccumulation of toxins through the diet. At the end of the bloom, decreased activity of antioxidant enzymes in the visceral and non-visceral tissues was detected in the bivalves, with an increase in oxidative damage (*p* < 0.05), in which the latter is correlated with the detection of the most toxic analogues of the STX-group (r = 0.988). In conclusion, in areas with high incidence of blooms, shellfish show a high activity of antioxidants, however, during the stages involving the distribution and bioconversion of toxins, there is decreased activity of antioxidant enzymes resulting in oxidative damage.

## 1. Introduction

Harmful algal blooms (HABs) are natural events that occur worldwide, which lead to significant economic, social, health, and tourism consequences, but due to their temporal variation in magnitude, the precise cost incurred by the affected countries is difficult to estimate [1,2]. Several biotic and abiotic factors have been noted as components that favor their incidence and prevalence in seas and freshwater streams. HABs are produced by dinoflagellates, diatoms, and cyanobacteria, which, by the increasing density of the microalgal community, may produce beneficial conditions for the development of aquaculture and marine life due to their role as primary producers in the aquatic ecosystem. However, in some species, these blooms are associated with the production of toxins which have different harmful effects on the ecosystem [3,4,5].

Around 100 species associated with HAB toxin production have been identified in the sea, where the saxitoxin-group (STX-group)-producing species have the highest prevalence and incidence worldwide [6,7]. This group of toxins is produced by marine dinoflagellates of the genera *Alexandrium*, *Pyrodinium* and *Gymnodinium* [8,9]. 

**Figure 1:**
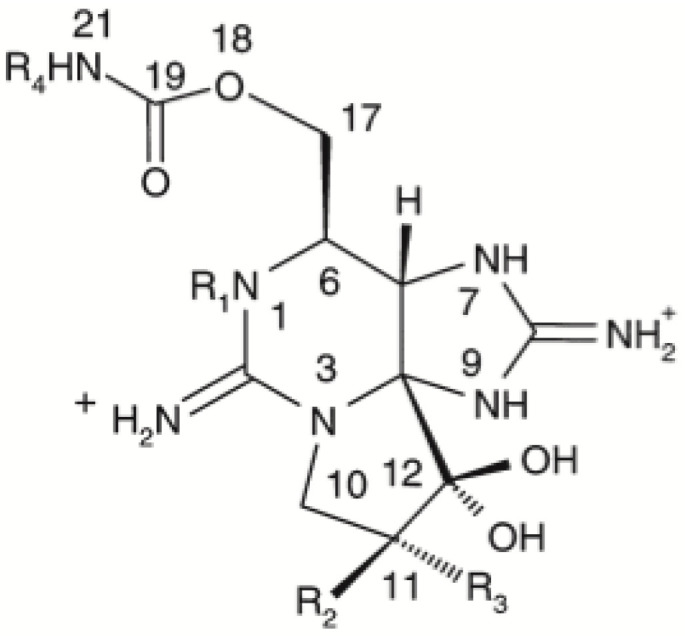
Chemical structures of saxitoxin (STX)-group toxins identified in contaminated dinoflagellates and molluscs. **R** indicates the substituted radical for each analogue.

STX-group toxins are made up of around 60 analogues which are characterized by having a central unit called an imidazoline that, according to the modification of some of its functional groups, can be divided into three groups: carbamoyl toxins including saxitoxin (STX), neosaxitoxin (neoSTX), gonyautoxin 4/1 (GTX4/GTX1) and gonyautoxin 3/2 (GTX3/GTX2); N-sulfocarbamoyl toxins (C1/C2, C3/C4) toxins and decarbamoyl toxins including decarbamoylsaxitoxin (dcSTX) decarbamoylgonyautoxin 4/1 (dcGTX4/dcGTX1) and decarbamoylgonyautoxin 3/2 (dcGTX3/dcGTX2), all of them analogues characterized by having different toxicities (toxic equivalent factor, TEF) [10,11] (Figure 1, Table 1).

These toxins have a blocking action on voltage-dependent sodium channels in nerve cells, preventing the transmission of the synaptic signal throughout the neuron, resulting in muscle paralysis, respiratory failure and, consequently, death [12,13].

The prevention of health risks, as well as the need for the industry to monitor the presence of toxins in several vectors, make it necessary to develop a surveillance program for potentially toxic microalgae. Several toxin detection methods have been developed to date, many of which allow the detection of toxins in shellfish well below maximum toxin levels. In most countries, the current regulatory standards on shellfish set the maximum accepted level of STX-group toxins in seafood at ≤80 μg of STX eq 100 g^−1^ tissue [14,15].

Filter feeding bivalves are the main vectors of STX-group toxins after filtering, accumulating, and biotransforming these toxins in their tissues and digestive glands. This allows these toxins to be transferred through the food chain to higher species such as carnivorous gastropods, fish, and marine mammals, which at the same time, leads to increased variability of the toxic profiles of toxins. In this way, the transfer of energy and toxins in the food chain may cause human poisoning through the consumption of contaminated seafood [12,16,17,18,19,20,21].

The prevalence of blooms associated with the production of STX-group toxins has made it possible to determine that shellfish constantly exposed to these toxins are more resistant if compared to shellfish not exposed to HABs, which, because of exposure, tend to reduce their feeding rates and consequently reduce the accumulation rates of toxins [22,23,24]. However, at the same time, the accumulated levels of toxins can produce a voltage-dependent block in sodium channels in the non-visceral tissues (foot and adductor muscle) of bivalves, leading to an alteration in the valve opening, leakage and paralysis, as well as stranding with subsequent decreased survival rate [9,25,26]. On the other hand, some species show greater variability in sensitivity towards STX-group toxins by favoring—through high filtration capacity—the accumulation of high concentrations of toxins in their tissues. This, in addition to the low depuration rate of some bivalve species, favors their ability to maintain toxicities in their tissues for a prolonged period of time (≤2 years old) [17,27,28].

In the southern pacific, STX-group toxins are produced by the species *Alexandrium pacificum*, whose blooms occur mainly between December and April, and where the influence of the oscillation of the ocean plays an important role by modifying the characteristics of the general circulation of continental waters and the temperature of the water column, either promoting or inhibiting the blooms of *Alexandrium pacificum* [29,30,31].

In Chile, in 2016, simultaneous HABs led to an estimated loss of USD 800 million, with a duration of approximately 8 months, significantly affecting myticulture (*Mytilus chilensis*) and the extraction of natural resources such as clams (*Ameghinomya antiqua*) and a gastropod called “loco” (*Concholepas concholepas*), both species having high commercial value [32,33,34]. In the same period, an unusual HAB expansion of the dinoflagellate *Alexandrium pacificum* was observed towards the oceanic zone of Chiloé Island, which was associated with the stranding of multiple hydrobiological species, including razor clam (*Mesodesma donacium*), with detected toxin levels > 8000 μg of STX eq 100 g^−1^ tissue [35]. However, in more southern areas of Chile, blooms are more prevalent, with repeated events of blooms of *Alexandrium pacificum* with no evidence of stranding or mortality of marine organisms (bivalves or gastropods), in which toxin levels > 143,000 μg of STX eq 100 g^−1^ in the tissue of mussels have been detected (*Mytilus chilensis*, Melchor Island, Aysén Region) [36].

Acute or chronic exposure to environmental pollutants, such as blooms associated with *Alexandrium pacificum* with the production of STX-group toxins, may produce a number of alterations in aquatic organisms. Although they can tolerate a certain number of short-term pollutant-induced biological disturbances, long-term exposure can deplete repair and defense mechanisms, causing a negative impact on cell organization, known as oxidative stress [37,38,39].

Oxidative stress is an important component of the biological response of marine organisms when exposed to a wide variety of environmental stressors on different scales of time and space [40]. Therefore, oxidative stress is produced when the rate of production of reactive oxygen species (ROS) exceeds the rate of elimination produced by endogenous antioxidant molecules [41,42]. Thus, this imbalance that leads to the production and accumulation of ROS causes damage to lipids, proteins, and the DNA of cells of aquatic organisms [43]. The damage can be determined depending on the species, age, organ, type of exposure, type of material, duration of the dose, and external environmental factors [44].

The antioxidant system is made up of substances that delay and/or prevent the oxidation of the cell substrate at low concentrations. This antioxidant defense system is widely distributed in living organisms and is extremely important due to its role in the direct elimination of free radicals [45]. The main enzymatic antioxidants are superoxide dismutase (SOD), catalase (CAT), and enzymes involved in the glutathione reductase (GR) metabolism process, glutathione transferase (GST) and glutathione peroxidase (GPx), which exert the primary defense against ROS [46,47].

In this way, the variability in the activity of enzymes that make up the antioxidant defense system can provide an early warning indicator of exposure to toxic compounds, allowing the identification of changes in biological systems before the effects are evident at the community level, such as the stranding or mass mortality of shellfish [48].

The objective of this research was to determine the variability of the antioxidant capacity according to the accumulation and biotransformation of toxins during a bloom of *Alexandrium pacificum* in mussels (*Mytilus chilensis*), clams (*Ameghinomya antiqua*), and the gastropod loco (*Concholepas concholepas*), endemic species of southern Chile.

## 2. Materials and Methods

### 2.1. Chemicals and Reagents

Acetic acid, acetonitrile, heptanesulfonic acid, hydrochloric acid, periodic acid methanol, nitric acid, and hydrogen peroxide were purchased from Merck (Merck, Darmstadt, Germany). Deionized water was obtained from a MicroPure water purification system (Thermo Scientific, Asheville, NC, USA). Chromatographic solvents were filtered through a membrane filter 0.45 μm from Merck (Merck Millipore Ltd., Cork, Ireland). The SOD, CAT, GPx, and GR assay kits were purchased from Sigma-Aldrich (Sigma-Aldrich; Merck KGaA, Darmstadt, Germany).

### 2.2. Standards

STX-group toxins quantification was assured using certified reference materials in solution (National Research Council Canada (Halifax, NS, Canada). To determine STX-group toxins, STX (CRM-STX-f), dcSTX (CRM-dcSTX), neoSTX (CRM-NEO-c), dcneoSTX (CRM-dcNEO), gonyautoxins (CRM-GTX2 and 3-c; CRM-GTX1 and 4-c; CRM-GTX5-b; CRM-dcGTX2 and 3-b), and C1–C2 (CRM-C1 and 2-b) were used. Stock solutions were diluted with acetic acid (500 mM) in order to obtain the appropriate work solutions. All solutions were stored in darkness at −20 °C.

### 2.3. Study Area and Sample Collection

**Figure 2:**
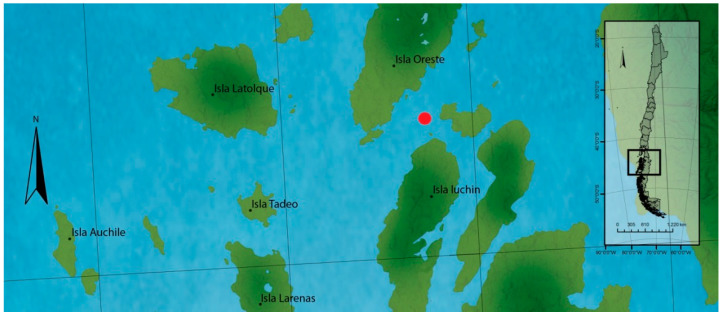
Sampling site for dinoflagellates, bivalves, and carnivorous gastropods near Huichas Island, Aysén Region, Chile.

To enable monitoring of the population dynamics of phytoplankton, a study site (S 45°02.040′ W 73°26.193′) was established near Huichas Island—Aysén Region (Chile)—during summer 2019 (Figure 2), under authorization No. 1238 of the Regional Secretariat of the Health Ministry, Region of Aysén del General Carlos Ibañez del Campo. Seawater sampling was carried out using Niskin bottles. Samples were collected daily (~1.7 L) every 1 metre along a vertical profile from the surface to the bottom (0–10 m depth) over a period of ≈70 days for monitoring algal bloom events. Water temperature and salinity were measured along a vertical profile at 0.5 m intervals from the surface to the bottom using a multiparameter probe (HI 9829, HANNA Woonsocket, RI, USA). For quantitative analysis of phytoplankton, 1.7 L water samples collected at each water depth were stored in polyethylene bottles and fixed with Lugol’s solution (2%). The fixed water samples were left to facilitate particle settling, then reduced to ≈10–50 mL by decanting the supernatant. The concentrated samples were loaded into a Sedgewick-Rafter counting chamber and cells of *Alexandrium pacificum* were counted using an inverted microscope (Nikon TE300, Nikon Instruments Inc., Melville, NY, USA). The results were expressed as mean ± standard deviation [49,50].

All samples, mussels (*Mytilus chilensis*), clams (*Ameghinomya antiqua*), and locos (*Concholepas concholepas*) were manually collected and kept at −20 °C until analysis. Sampling depths varied between 1 and 10 m, but most samples were taken within the 2–5 m depth stratum. For the analyses, samples representative of each species were considered. Each sample included about 100 individuals per species [51]. The results were expressed as mean ± standard deviation.

### 2.4. STX-Group Sample Preparation

The experiments in this project were approved by the Faculty of Medicine, Universidad de Chile, Bioethical Committee (CBA 0862 FMUCH), and the Institutional Biosafety Committee project (Project Nº1160168). Live species were collected at Huichas Island and upon arrival at the laboratory, samples were extracted from shellfish separately, and 100 g of visceral (digestive glands) and non-visceral (foot and gill) tissue was removed to determine the toxin level in each species. The shellfish samples were then transferred to 250 mL centrifuge tubes with the same volume of 0.1 N HCl and homogenized; the toxins present in the samples were extracted following the AOAC procedure [52]. All samples were carefully treated to avoid variations in the profile of toxins produced by changes in pH. Small aliquots were taken to quantify the toxin concentration of the extracts by HPLC. Materials used during the experimental work were disposed of according to the protocols for chemical and biological waste disposal of the Biosafety Unit of the Faculty of Medicine of the Universidad de Chile.

### 2.5. High-Resolution Liquid Chromatography with Fluorescent Detection (LC-PCOX)

Detection of STX-group toxins was accomplished by using the LC-PCOX AOAC 2011.02 technique [12,53]. An HPLC unit (Young Lin Instrument, Co., Anyang, Korea) was used, equipped with a binary pump (YL9101) at a constant flux of 0.8 mL/min of the mobile phase, with a Rheodyne 7725i coupled to a spectrofluorometer (FP-2020 Plus, Jasco, Tokyo, Japan), in an excitation range of 330 nm and an emission range of 390 nm. To determine carbamate toxins (GTXs and STX), a 3.5 μm reverse phase C-8 column (Zorbax Bonus-RP, 4.6 × 150 mm, Agilent Technologies Co., Ltd., Santa Clara, CA, USA) was used and, to determine sulfocarbamoyl toxins (C1/C2) a 5 μm reverse phase C-8 column (BetaBasic-8, 4.6 × 250 mm, Fisher Scientific, Nepean, ON, Canada) at constant 37 °C (column compartment YL 9131, YL Instrument Co., Ltd. Gyeonggi-do, Korea) was used. Besides the LC binary pump, two additional isocratic pumps (YL9200) (YL Instrument Co., Ltd. Gyeonggi-do, Korea) were used, one with an oxidant agent and the other with 0.75 M nitric acid, with a flux of 0.4 mL/min. All elements were lined up inside a reaction oven at 85 °C (CO-IV Scienhome, Scienhome Scientific Instrument Co. Ltd., Tianjin, China), which contained 10 m of coiled peek tubing with a total volume of 1 mL for derivatization of toxins. All toxins were identified comparing their retention time (*R_t_*) measured as min/V. Quantification of each analogue was done according to its 0.01 to 4.5 μg g^−1^ interval of STX-equivalent (*r*^2^ = 0.9989) calibration curve. LOD and LOQ of STX-equivalents were calculated according to IUPAC criteria, establishing a range between 0.005 to 0.02 μg g^−1^ and 0.01 to 0.2 μg g^−1^ respectively [12,54]. Total toxicities of species were expressed as μg STX-equivalent 100 g^−1^, utilizing the TEF of each toxin [14].

### 2.6. Preparation of the Tissues

The species were dissected according to the type of tissue to be analyzed. From mussels and clams the digestive glands, gills, and feet were obtained, and from the gastropods, feet and digestive glands were obtained. 

Tissues of each species were collected separately and homogenized on ice in a TRIS buffer (100 mM, pH 7.8; 1:10 *w*/*v*) using an Ultra-Turrax^®^. After centrifuging at 21,000× *g* for 5 min at 4 °C, the resulting supernatant was diluted (10% *v*/*v*) and used to determine the activities of the enzymes SOD, CAT, GPx, and GR [55]. The enzymatic parameters were analyzed in quintuplicate using appropriate reagents (Sigma Aldrich, St. Louis, MO, USA).

### 2.7. Antioxidant Defenses

To determine superoxide dismutase (SOD) activity, we followed the method described by Simos et al. [56]. Along with 50 μL of freshly prepared supernatant obtained from the tissue of each species, 1.3 mL of WST-1 ([2-(4-iodophenyl)-3-(4-nitrophenyl)-5-(2,4- disulfophenyl)-2H- tetrazolium, monosodium salt]) was incubated. The reaction was initiated by the addition of 10 μL xanthine oxidase (50 μM), and incubated for 20 min at 37 °C, then the absorbance at 450 nm was determined (Biotek Synergy HT Multi-mode microplate reader). One unit of SOD was defined as the amount of enzyme needed to exhibit 50% dismutation of the superoxide radical and was expressed as units (U)/mg total proteins (U/mg).

To determine catalase (CAT) activity, we followed the method described by Kwok et al. [57]. Supernatant (50 μL) from each tissue type (digestive gland, gill, and foot) was supplemented with 1.1 mL of 500 mM potassium phosphate buffer (pH 7.0) at 25 °C, and then 20 μL of H_2_O_2_ (20 mM) was added. The reaction was mixed and incubated for 5 min at 25 °C. Then, the reaction was stopped with the addition of 15 μL of sodium azide (15 mM). From the final volume, 10 μL were taken and mixed with 1.0 mL of potassium phosphate buffer (150 mM, pH 7), containing 0.25 mM 4-aminoantipyrine and 2 mM 3.5-dichloro-2-hydroxybenzenesulfonic acid, and then incubated for 15 min at 25 °C. The final reaction was analyzed at 520 nm absorbance (Biotek Synergy HT Multi-mode microplate reader). The values of catalase activity are expressed as units (U)/mg protein. One unit of catalase is responsible for the consumption of 1 mol of H_2_O_2_ per minute.

To determine glutathione reductase (GR) activity, we followed the method described by Han and Han [58]. GR activity was measured by incubating 2 mM of oxidized glutathione in 100 mM potassium phosphate buffer, with 1 mM EDTA and 0.1 mM KCl (pH 7.5). Then, 30 μL freshly prepared supernatant from each tissue sample (bivalve or gastropod) was added followed by 50 μL NADPH (2 mM). After 5 min preincubation (37 °C), the reaction was initiated by the addition of 100 μL GSSG (1 mM). The final reaction volume was analyzed at 340 nm absorbance (Biotek Synergy HT Multi-mode microplate reader). The values of GR activity are expressed as μmol/mg protein.

To determine glutathione peroxidase (GPx) activity, we followed the method described by Gupta and Baquer [59]. GPx activity was determined by incubating 930 μL Tris HCl (50 mM), containing 0.5 mM EDTA (pH 8), 50 μL NADPH (5 mM), 50 μL reduced glutathione (42 mM), 10 units/mL of glutathione reductase, and 50 μL supernatants from each tissue sample of the bivalves and gastropods. After 5 min of preincubation at 25 °C, the reaction was initiated by the addition of 10 μL tert-butyl-hydroperoxide (30 mM). The final reaction was analyzed at 340 nm absorbance (Biotek Synergy HT multi-mode microplate reader). The values of GPx activity are expressed as μmol/mg protein.

### 2.8. Protein Oxidation

To determine the carbonyl content, we followed the method described by Parraguez et al. [60]. The supernatant from each tissue sample (bivalve or gastropod) was centrifuged at 21,000× *g* for 5 min at 4 °C and the resulting volume was incubated for 15 min with 10% streptomycin sulphate at 4 °C to eliminate DNA debris. From the resulting mixture, 100 μL of supernatant and 100 μL of fresh 2,4-dinitrophenylhydrazine (DNPH, 10 mM) prepared in 2M HCl was added. The contents were mixed and incubated in the dark at room temperature for 10 min. After the time had elapsed, 30 μL of 100% trichloroacetic acid (TCA) (*w*/*v*) was added and the tube was incubated for 15 min on ice, to be then centrifuged at 7200× *g* for 15 min at 4 °C to collect the protein pellet. The supernatant was aspirated and discarded. The precipitates were dissolved in 200 μL 6 M guanidine hydrochloride and incubated at 37 °C for 10 min. The insoluble materials were removed by centrifugation (11,000× *g* for 3 min at 4 °C) and 100 μL was used to determine the content of carbonyl at 375 nm absorbance. Results are expressed as μmol carbonyl per mg protein.

### 2.9. Lipid Peroxidation

To determine the lipid peroxidation (LPO) content, we followed the method described by Zeb and Ullah [61]. The tissue from each sample of the bivalves and gastropods (0.5 mL) was homogenized in an ice bath with 30 μL of butylated hydroxytoluene (BHT) 1% mass/vol in glacial acetic. Then, the samples were centrifuged at 21,000× *g* for 10 min at 4 °C. From the supernatant, a sample of 200 μL was used and 600 μL thiobarbituric acid (TBA, 1%) was added. The mixture was then heated for 60 min at 95 °C. Subsequently, the reaction was cooled in an ice bath for 10 min. Of this reaction, 200 μL was used to determine the content of TBA at 532 nm absorbance. TBARS concentrations were derived from an external standard curve of 1,1,3,3-tetramethoxypropane (malondialdehyde; MDA). Lipid peroxidation content was calculated as μmol TBA reactive substance (μmol TBARS)/mg protein.

### 2.10. Protein Concentration

Protein concentration was determined using the Bradford method (Bio-Rad, Protein assay, Hercules, CA, USA) [62]. The standard curve was generated using bovine serum albumin (BSA) as standard.

### 2.11. Statistical Analyses

Results were expressed as mean ± SEM (*n* = 5). Calibration curves were obtained through regression analyses. Differences between groups were analyzed using one- or two-way analysis of variance (ANOVA) depending on the number of variables to be analyzed and Dunnett’s test as a *post hoc* test. A *p* < 0.05 significance level was considered for all cases. Analyses were performed using the GraphPad Prism software (GraphPad Prism 7, GraphPad Software, Inc., La Jolla, CA, USA).

## 3. Results

### 3.1. Toxin Composition in Dinoflagellate and Accumulation of Toxins in Shellfish Species

**Figure 3:**
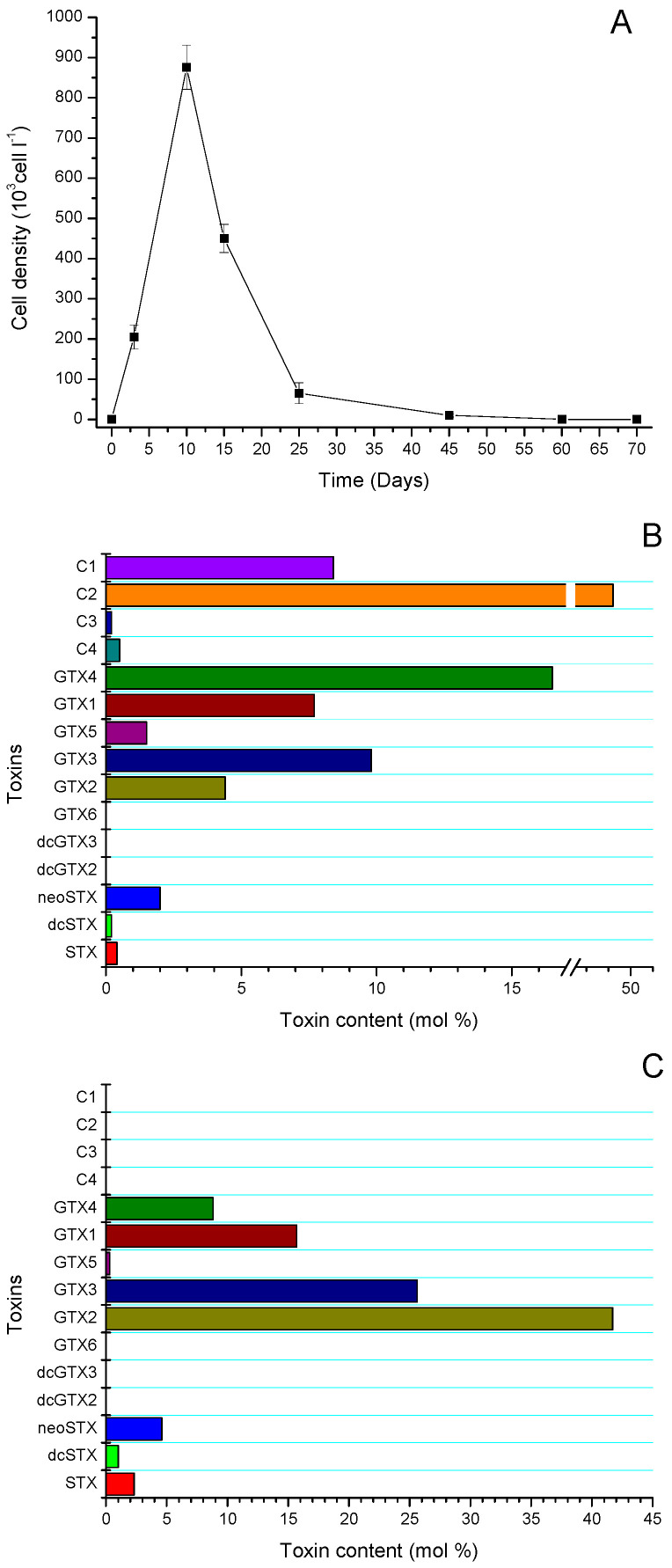
(**A**) Variation in cell concentration (cell/L), and changes in the toxin profile during the (**B**) exponential phase [12], and (**C**) decrease of a bloom of *Alexandrium pacificum* near Huichas Island, Aysén Region, Chile.

Species were collected in the study area of the Huichas Islands zone (Figure 2) which is characterized by a high incidence of blooms associated with *Alexandrium pacificum*. In the study area, a local bloom associated with the microalgae was detected, with a maximum density of ≈890 ± 45 × 10^−3^ cell/L (day 10), whose toxicity was found to be 22.7 pg STX-equiv/cell (Figure 3A). The toxins identified in the microalgae in the exponential phase included: C1 (8.4%), C2 (48.4%), C3 (0.2%), C4 (0.5%), GTX4 (16.5%), GTX1 (7.7%), GTX5 (1.5%), GTX3 (9.8%), GTX2 (4.4%), neoSTX (2.0%), dcSTX (0.2%), and STX (0.4%). No carbamoyl derivatives were detected (dcGTX 2 and dcGTX 3) (Figure 3B). The bloom had a duration of ≈25 days due to the environmental instability of the area. During the bloom-decay period, the toxins identified in the microalgae included: GTX4 (8.8%), GTX1 (15.7%), GTX5 (0.3%), GTX3 (25.6%), GTX2 (41.7%), neoSTX (4.6%), dcSTX (1.0%), and STX (2.3%) (Figure 3C).

**Figure 4:**
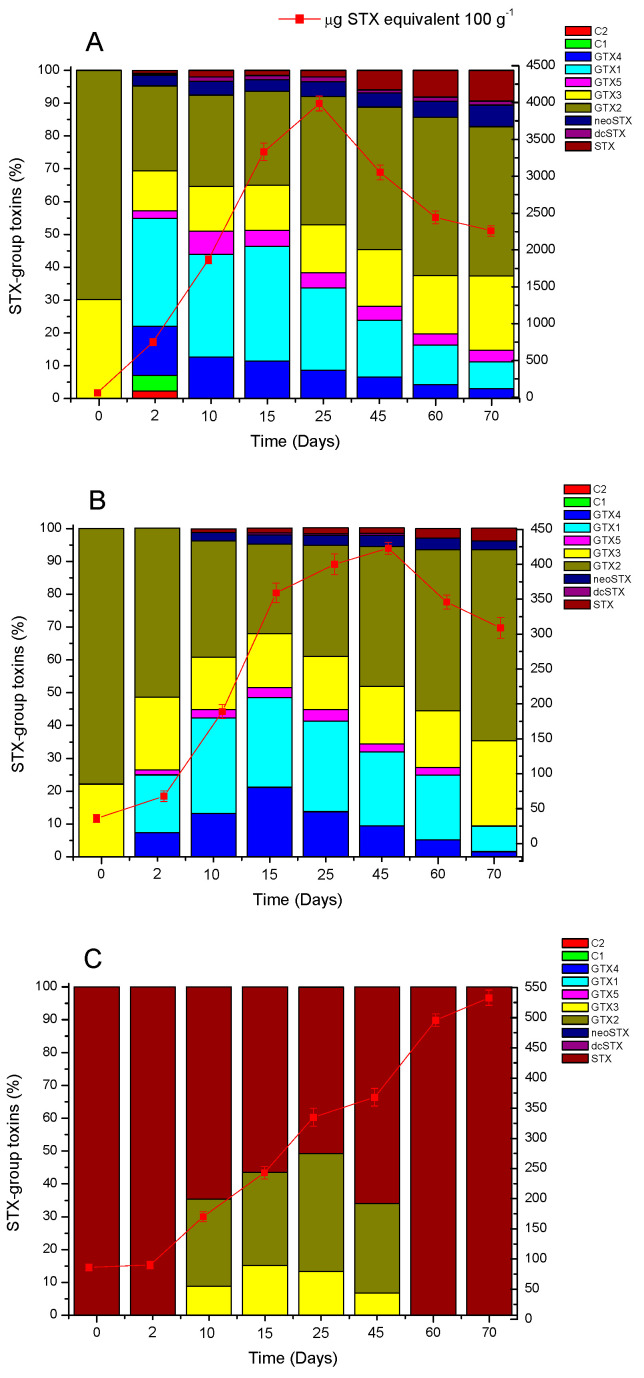
Total STX-group toxicity (μg STX equivalent 100 g^−1^) and changes in the toxin profile (%) in digestive glands of (**A**) mussels (*Mytilus chilensis*), (**B**) clams (*Ameghinomya antiqua*), and (**C**) feet of locos (*Concholepas concholepas*) during a bloom of *Alexandrium pacificum*. Data are presented as the mean ± standard deviation (SD). (*n* = 5).

Before (day 0), during (days 2, 10, 15, and 25) and after the bloom (days 45, 60, and 70), toxin evaluations were carried out in mussels (*Mytilus chilensis*), clams (*Ameghinomya antiqua*), and locos (*Concholepas concholepas*), which are the most prevalent species in the area. During this period, changes were detected in the toxin content of all species (Figure 4). Before the bloom of *Alexandrium pacificum* (time point 0), mussels, clams, and locos showed a baseline toxicity of 53.2 ± 3.2; 36.7 ± 1.3 and 86.5 ± 2.4 μg STX equiv 100 g^−1^, respectively. In mussels and clams, the most prevalent toxins identified at time point 0 were GTX3/GTX2, while in locos, only STX was detected.

Once the bloom was detected, mussels showed a significant increase in toxicity with a maximum on day 25, while in clams, a maximum toxicity was detected on day 45. The frequency of the toxic analogues identified in mussels and clams during the 70 days of analysis is shown in Figure 4A,B. In turn, locos showed a maximum toxicity 45 days after the end of the bloom (Figure 4C).

On day 10, mussels showed a toxicity of 1788 ± 10.7 μg STX equiv 100 g^−1^, with a toxin composition including GTX4 (12.7%), GTX1 (31.2%), GTX5 (7.1%), GTX3 (13.6%), GTX2 (27.8%), neoSTX (4.2%), dcSTX (1.4%), and STX (2.0%). Maximum toxicity was detected on day 25, with 3988.5 ± 9.2 μg STX equiv 100 g^−1^, and a toxin composition including GTX4 (8.6%), GTX1 (25.1%), GTX5 (4.6%), GTX3 (14.6%), GTX2 (38%), neoSTX (4.6%), dcSTX (1.0%), and STX (3.5%). While in the final stage of the evaluation (day 70), a toxicity of 2227.4 ± 13.6 μg STX equiv 100 g^−1^ was detected, with a toxin composition including GTX4 (3.0%), GTX1 (8.2%), GTX5 (3.5%), GTX3 (22.7%), GTX2 (45.4%), neoSTX (6.6%), dcSTX (1.2%), and STX (9.4%) (Figure 4A).

In clams, after 10 days, a toxicity of 189.1 ± 3.7 μg STX equiv 100 g^−1^ was detected, with a toxin composition including GTX4 (13.2%), GTX1 (29.1%), GTX5 (2.6%), GTX3 (15.9%), GTX2 (35.4%), neoSTX (2.6%), and STX (1.1%). The maximum toxicity was detected on day 45, with 423.4 ± 1.8 μg STX equiv 100^−1^ g, with a toxin composition including GTX4 (9.5%), GTX1 (22.5%), GTX5 (2.4%), GTX3 (17.5%), GTX2 (42.6%), neoSTX (3.5%), dcSTX (0.5%), and STX (1.7%). At the final stage of the analysis (day 70), a toxicity corresponding to 309.3 ± 5.1 μg STX equiv 100 g^−1^ was detected in clams, with a toxin composition including GTX4 (1.6%), GTX1 (7.8%), GTX3 (25.9%), GTX2 (58.3%), neoSTX (2.6%), and STX (3.9%) (Figure 4B).

In addition, before the bloom, locos showed a toxicity of 86.5 ± 2.4 μg STX equiv 100 g^−1^, with a toxin composition including only STX. The toxicity of the species progressively increased with a toxicity of ≈350.3 ± 4.4 μg STX equiv 100 g^−1^ (day 25) with a toxin composition including GTX3 (25.9%), GTX2 (13.4%), neoSTX (35.8%), and STX (50.7%). The gastropod attained a maximum toxicity of ≈533.7 ± 3.8 μg STX equiv 100 g^−1^ 70 days after the detection of the bloom, with a toxin composition including only STX (Figure 4C).

### 3.2. Antioxidant Enzyme Levels and Oxidative Damage in Shellfish Species during and after Bloom of Alexandrium pacificum

Regarding the content of toxins assimilated by mussels, clams, and locos, they were collected over 70 days (during and after the algal bloom). In these species, activities of the enzymes SOD, CAT, GPx, and GR were analyzed, as well as carbonyl contents and lipid peroxidation in the digestive glands, gills, and feet of mussels and clams, and in the viscera and feet of locos. Our objective was to correlate the responses of enzyme activities and oxidative damage with the level of assimilation and distribution of toxins, as well as the variation of the frequency of STX-group analogues which resulted from the biotransformation processes produced in the visceral and non-visceral tissues of the species.

#### 3.2.1. Superoxide Dismutase Activity

**Figure 5:**
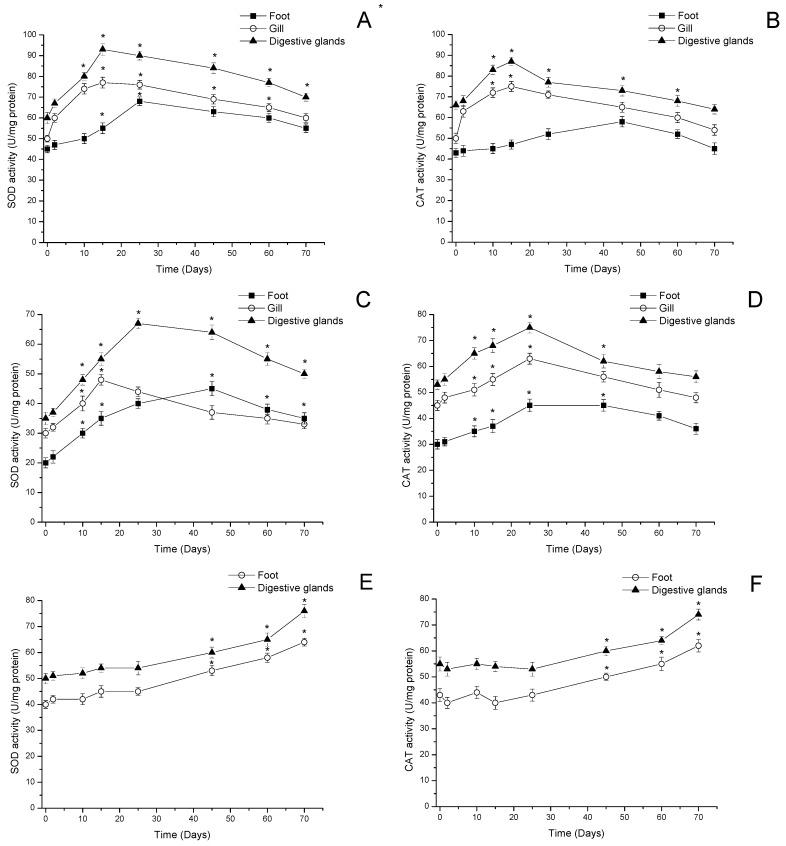
SOD and CAT activities in feet, gills, and digestive glands of (**A**,**B**) mussels (*Mytilus chilensis*), (**C**,**D**) clams (*Ameghinomya antiqua*), and (**E**,**F**) feet and digestive glands of locos (*Concholepas concholepas*) during a bloom of *Alexandrium pacificum*. Data are presented as the mean ± SEM. For significant differences compared to control (time point 0), values are indicated by an asterisk (*) in the data (*p* < 0.05; Dunnett’s test).

In Figure 5A, the variation of superoxide dismutase (SOD) activity in mussels is shown. SOD in digestive glands and gills showed increased activity from day 2 of the bloom with a maximum at day 15 (>50 and 56%, in relation to day 0, *p* < 0.05) which was correlated with the amount of toxin detected (r = 0.897). Subsequently, a gradual decrease was detected in activity from day 25 to day 70 by approximately 20% in gills (≈58 Umg/protein, *p* < 0.05) and 24% in digestive glands (≈70 Umg/protein, *p* < 0.05) in relation to the maximum level of activity detected. In the feet, maximum SOD activity was detected 25 days after the onset of the bloom (>44%, ≈65 Umg/protein, *p* < 0.05). SOD activity began to decrease after day 25 until day 70, when a decrease in SOD activity ≈15% (≈55 Umg/protein) was detected in relation to the maximum activity determined (Figure 5A).

In clams, the activity of SOD increased significantly depending on the concentration of toxins assimilated in the digestive glands and the toxins compartmentalized in the feet (>15 days, *p* < 0.05) (Figure 5C). At time point 0 (before the beginning of the bloom), baseline SOD activity detected in the feet and digestive glands was ≈30.2 Umg/protein and 35 Umg/protein, respectively, which gradually increased over time. The maximum activity detected for gills and digestive glands was on day 15 and 25, respectively, with a maximum of ≈60% and ≈114%, in relation to time point 0 (*p* < 0.05). SOD activity began to decrease from day 25 in gills and day 45 in digestive glands, reaching a basal activity at day 70 of ≈30 Umg/protein and 50 Umg/protein, respectively. In the feet, SOD activity gradually increased until it reached a maximum activity 45 days after the beginning of the bloom (>110%, ≈44 Umg/protein, *p* < 0.05), and then activity began to decrease until day 70, when activity higher than day zero pre-exposure to bloom was determined (Figure 5C).

In locos, an increase in SOD activity was detected in feet and viscera in relation to the concentration of toxins detected. The baseline activity at time point 0 in feet and viscera was ≈41.3 Umg/protein and ≈50.7 Umg/protein, respectively, which was increasing significantly in relation to the control (time point 0), until day 70 when SOD activity in feet and viscera was ≈60.5 Umg/protein and 74.2 Umg/protein (*p* < 0.05), respectively (Figure 5D). The increase in activity was significant (*p* < 0.05) at all analysis time points in relation to the control (time point 0).

#### 3.2.2. Catalase Activity

In Figure 5B,D,F, the variations of catalase (CAT) activity in mussels, clams, and locos during the bloom period are shown. In mussels, the increase in activity was significant at time points evaluated (days 10 and 15, *p* < 0.05) in gills and digestive glands, and a maximum activity in relation to day 0 of ≈48% was detected in gills (74.4 Umg/protein, *p* < 0.05) and 30% in digestive glands (85.1 Umg/protein, *p* < 0.05). CAT activity subsequently began to decrease gradually until day 70, when activities similar to time point 0 were detected in both tissues. In the mussels’ feet, a progressive increase in CAT activity was detected from day 0 until day 25; the maximum activity was detected at day 45 if compared to the day of the beginning of the bloom (55.5 Umg/protein, *p* < 0.05). Subsequently, CAT activity in the feet began to decrease until day 70 when activities similar to time point 0 were detected (Figure 5B).

In clams, CAT activity showed similar trends in the assessed visceral and non-visceral tissues (feet, gills, and digestive glands), with a progressive increase in the activity from the beginning of the bloom until reaching a maximum of activity on day 25, with a significant increase in activity in relation to day 0 of ≈44% in digestive glands, ≈33% in gills, and ≈46% in feet (*p* < 0.05). Subsequently, CAT activity in all tissues began to progressively decrease to the activity levels detected before the bloom (Figure 5D).

In turn, CAT activity in the gastropod loco showed a similar trend in feet and viscera, with a significant increase 70 days after the beginning of the bloom, when the maximum activity was detected (≈36%, 60.7 Umg/protein in feet and ≈34%, 74.4 Umg/protein in viscera, *p* < 0.05) (Figure 5F).

#### 3.2.3. Glutathione Peroxidase and Glutathione Reductase Activity

**Figure 6:**
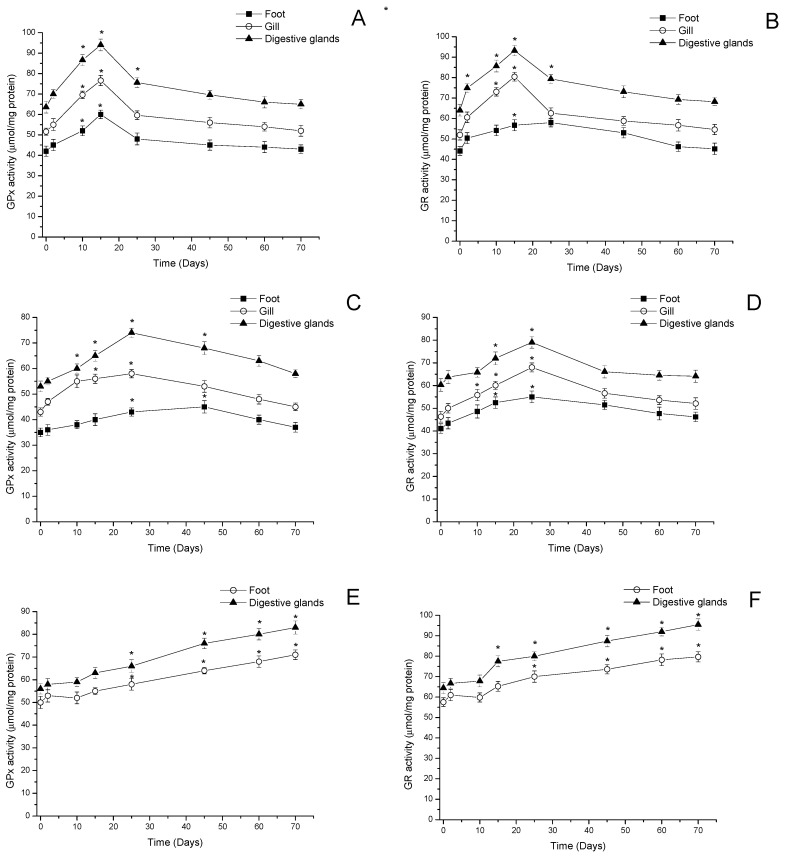
GPx and GR activities in feet, gills, and digestive glands of (**A**,**B**) mussels (*Mytilus chilensis*), (**C**,**D**) clams (*Ameghinomya antiqua*), and (**E**,**F**) feet and digestive glands of locos (*Concholepas concholepas*) during a bloom of *Alexandrium pacificum*. Data are presented as the mean ± SEM. For significant differences compared to control (time point 0), values are indicated by an asterisk (*) in the data (*p* < 0.05; Dunnett’s test).

In relation to activities of glutathione peroxidase (GPx) and glutathione reductase (GR), they showed significant changes according to the analysis time points in the visceral and non-visceral tissues of mussels, clams, and locos (Figure 6).

In the feet, gills, and digestive glands of mussels, GPx levels increased significantly from day 10 (≈25% in feet, ≈36% in gills, and ≈34% in digestive gland, *p* < 0.05), reaching maximum activity at day 15 (≈31% in feet, ≈50% in gills, and ≈46% in digestive glands, *p* < 0.05) (Figure 6A). In GR, a similar activity was determined in gills and digestive glands with a maximum activity level 25 days after the beginning of the bloom (≈50% in gills and ≈45% in digestive glands, *p* < 0.05). However, the feet of the mussels showed a slight increase in GR activity on day 25, which is not significant. At the end of the period, all tissues showed a GR activity similar to that at time point 0 (Figure 6B).

In clams, maximum GPx and GR activities were registered on day 25 after the beginning of the bloom with an increase of ≈25% in gills and ≈37% in digestive glands (*p* < 0.05) for GPx, and an increase of ≈44% in gills and ≈28% in digestive glands (*p* < 0.05) for GR (Figure 6C,D). In the feet, GPx activity showed increased activity with a maximum on day 45 after the beginning of the bloom (≈21%, *p* < 0.05); the maximum GR activity recorded was on day 25 with an increase in relation to time point 0 of ≈32% (*p* < 0.05). Seventy (70) days after the beginning of the bloom, GPx and GR activities decreased to values similar to those detected at time point 0 (Figure 6C,D).

In the gastropod loco, GPx activity in feet and viscera showed a variation (≈10%) after 25 days, which increased significantly on day 45 to ≈20% (55.1 and 64.6 μmol/mg protein respectively, *p* < 0.05), reaching maximum activity on day 70 (65.1 and 79.6 μmol/mg protein, respectively, *p* < 0.05) (Figure 6E). Moreover, GR activity showed changes in the feet and digestive glands of locos; in both tissues, the increase in GR activity was significant on day 45 with respect to the control (*p* < 0.05), and maximum activity on day 70 in relation to the control was found (>16% in feet and 46% in digestive glands, *p* < 0.05) (Figure 6F).

#### 3.2.4. Oxidative Damage

**Figure 7:**
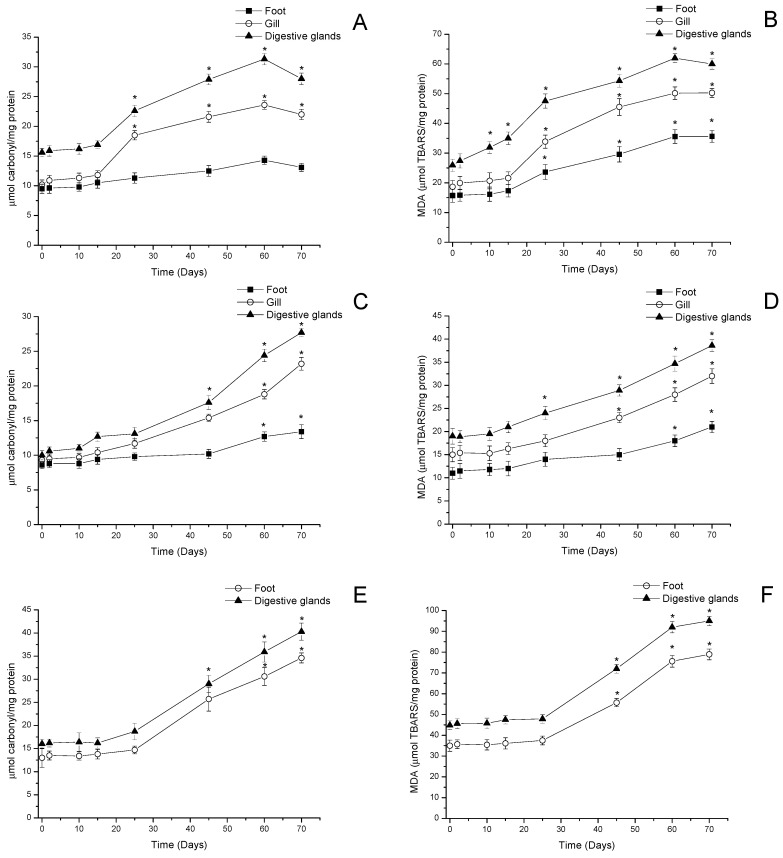
Protein oxidation (carbonyl content) and lipid peroxidation (MDA) in feet, gills, and digestive glands of (**A**,**B**) mussels (*Mytilus chilensis*), (**C**,**D**) clams (*Ameghinomya antiqua*), and (**E**,**F**) feet and digestive glands of locos (*Concholepas concholepas*) during a bloom of *Alexandrium pacificum*. Data are presented as the mean ± SEM. For significant differences compared to control (time point 0), values are indicated by an asterisk (*) in the data (*p* < 0.05; Dunnett’s test).

Oxidative damage in visceral and non-visceral tissues in mussels, clams, and locos was determined by protein oxidation (carbonyl groups) and lipid peroxidation (MDA). In mussels, oxidative damage showed an increase 25 days after the beginning of the bloom in carbonyl content in digestive glands (≈66%, *p* < 0.05) and gills (≈60%, *p* < 0.05) reaching a peak on day 60 with an increase of ≈140% in gills and ≈100% in digestive glands (*p* < 0.05). After 10 days, a decrease in protein oxidation (carbonyl groups) of ≈40% was detected in gills and digestive glands (*p* < 0.05). In feet, no significant variations during the 70 days of analysis were detected (Figure 7A). In addition, the MDA content showed a similar trend among mussel tissues, with a progressive increase in MDA content between 33% and 80% on day 25 (*p* < 0.05), reaching a maximum peak in all tissues on day 60 (>100%, *p* < 0.05) (Figure 7B).

In clams, carbonyl content showed a significant increase in gills and digestive glands from day 45 onwards if compared to time point 0 (≈50% and ≈60% respectively, *p* < 0.05) reaching a peak in both tissues after 70 days (≈150% and ≈180% respectively, *p* < 0.05). Furthermore, the feet of the clam showed increased carbonyl content at 60–70 days >20% if compared to time point 0 (Figure 7C). MDA levels in this species showed a correlation with those detected in carbonyl content (r = 0.957), showing a progressive increase from day 45 in digestive glands (≈57%, *p* < 0.05), and gills (≈46%, *p* < 0.05), while in feet, the MDA increase was detected on day 60 (*p* < 0.05). All tissues reached a maximum level of MDA content after 70 days (≈100% in digestive glands, gills, and feet *p* < 0.05) (Figure 7D).

Regarding the gastropod loco, the carbonyl and MDA content showed a progressive increase in all tissues (feet and viscera) from day 45 (>80% if compared to time point 0, *p* < 0.05) reaching a maximum peak at 70 days (>140% if compared to time point 0, *p* < 0.05) (Figure 7E,F).

## 4. Discussion

Marine ecosystems are subject to constant environmental changes related to anthropogenic activities and climate change, so primary producers such as phytoplankton tend to be the first to capture environmental variables, producing in some cases an increase in their biomass, which can lead to different effects throughout the food web [63].

*Alexandrium pacificum* is the most prevalent species in the Southern Pacific area of Chile, characterized by producing blooms associated with the production of STX-group toxins [64]. These blooms are generally favored or limited by the stoichiometric relationships between biotic and abiotic components that allow for bloom expansion or inhibition. Thus, it is possible to understand the different oceanographic expansions that take place each year in the different fjords and channels of southern Chile associated with this species [3,12]. The above was evidenced in 2016 by the first oceanographic extension of an algal bloom associated with *Alexandrium catenella* towards the coast of Cucao (Chiloé, Chile), where a massive beaching of surf clams *Mesodesma donacium* was produced (toxicity > 8000 μg STX equiv 100 g^−1^). The results indicated that such environmental damage was the result of an indirect effect of STX-group toxins, and of inflammatory responses detected in the digestive glands [33,35].

In the southern fjords, the different marine species, to some degree, tend to be exposed to algal blooms associated with *Alexandrium pacificum*, by assimilating, bioaccumulating, and biotransforming the toxins produced by this dinoflagellate. However, since blooms depend on environmental conditions, microalgae tend to migrate in the water column. Therefore, the assimilation and bioaccumulation of toxins tend to be species-specific, as well as the bioconversion capacity of toxins, which depends largely on the enzymatic activity characteristic of each species [17,49].

In this study, the detection of a bloom of *Alexandrium pacificum* in the area of Huichas Islands (Aysén Region) allowed us to determine changes in antioxidant enzyme activities and oxidative damage in three endemic species from southern Chile. We were also able to correlate these activities with the degree of toxin assimilation both directly and through the trophic chain.

During 2019, in the area of Huichas Islands, Aysén Region, a bloom of *Alexandrium pacificum* was detected, which lasted for approximately 25 days, the maximum cell density was ≈890 × 10^3^ cell/L. After 15–20 days, the bloom decreased to levels of <1−10 × 10^3^ cell/L. The exponential phase of the bloom was characterized by being associated with a toxin profile dominated by *N*-sulfocarbamoyl toxins (C2/C1, ratio 4:1) and carbamoyl toxins (GTX4/GTX1 and GTX3/GTX2, ratio between epimers 2:1). The profile of toxins detected was similar to those previously detected in the area; however, it has been established that *Alexandrium* sp. can produce blooms with very different dynamics and toxicities in different regions, which could be connected to a specific response to the environmental conditions to which the microalgae are exposed, which tends to be characteristic in the southern fjords [64]. Therefore, it is possible to explain the variation of the toxin profile detected in the senescent phase of the bloom, in which carbamates GTX3/GTX2 were mostly detected with a preferential ratio to α-epimers.

During this period, the toxin profile and toxicity of the most prevalent resources in the area were also analyzed, corresponding to mussels (*Mytilus chilensis*), clams (*Ameghinomya antiqua*) and the gastropod loco (*Concholepas concholepas*). *Mytilus chilensis* is a species characterized by an epifaunal habitat and by being attached to a rocky substrate in the medium to high rocky shore zone [22]. It feeds by pumping seawater past its gills to filter out food items and other suspended particles. Then, they are bioaccumulated into its tissues or passed through its bodies undigested [17,49]. Before the bloom, this species showed a toxicity of ≈53.2 ± 3.2 μg STX equiv 100 g^−1^ with a profile dominated by GTX3/GTX2 (ratio 1:3), and then, from the second day after the beginning of the bloom, progressive increases in toxicity were observed, reaching ≈3988 μg STX equiv 100 g^−1^ (*p* < 0.05). This increased toxicity was added to the high bioconversion of the analogues in relation to those detected in the dinoflagellate, preferentially towards more thermostable toxic forms via hydrolysis (C2/C1→GTX3/GTX2), epimerization (GTX4→GTX1; GTX3→GTX2), and reduction (GTX4/GTX1→neoSTX; GTX3/GTX2→STX), in which no specific accumulation of toxins was detected [28,65]. The habitat of *Mytilus chilensis,* added to its direct assimilation of HABs and its high filtration rate, have made it an excellent bioindicator of toxicity in the region. However, the interaction with repeated blooms of *Alexandrium* sp. produce an initial decrease in the filtration capacity of the dinoflagellate in this species, a process that is reversible as the bloom increases its exponential phase, suggesting, therefore, an acclimatization when interacting with the toxic algae [66]. This could explain the high toxicities recorded in the area of the southern fjords, reaching values of >100,000 μg STX equiv 100 g^−1^ and at the same time, its high bioconversion capacity, which broadens the toxin profile without causing mortality of the species [17].

*Ameghinomya antiqua* is a bottom-dwelling species with a diet associated preferentially with sediments, organic detritus, and the assimilation of cells associated with blooms, mostly senescent cells and/or those rejected from other marine organisms [17,67]. The initial toxicity in *Ameghinomya antiqua* was 36.7 ± 1.3 μg STX equiv 100 g^−1^ with a prevalence of the analogues GTX3/GTX2 (ratio 1:5). On the second day of exposure to bloom, its toxicity reached ≈76.1 ± 2.2 μg STX equiv 100 g^−1^ with a profile characterized by GTX4/GTX1, GTX5, and GTX3/GTX2. Subsequently, toxicity gradually increased until it reached a peak on day 45 with a toxicity of ≈423 ± 1.8 μg STX equiv 100 g^−1^ (*p* < 0.05), with a profile characterized by α-epimers (GTX1 and GTX2) and carbamates, mainly neoSTX and STX. This species is characterized by showing low toxicities if compared to other species, such as mussels, which are associated with a more rapid accumulation of toxins in their tissues [12,17,49]. Previous studies have shown that sandy bottom species may survive in an environment subjected to HABs by producing an increase in sediment depth in an attempt to avoid direct interaction with the toxic bloom as a response [68]. In addition, it has been determined that some types of clams, when exposed to HABs, tend to vary their valve opening times, which would allow them to decrease the filtration rate [69]. This action of reducing the valve opening is often accompanied by retraction of the mantle edges and siphons, with a high production of pseudofaeces, a characteristic process during episodes of HABs and/or when subjected to environmental stress conditions [42,70,71].

During the bloom period associated with *Alexandrium pacificum*, the activity of antioxidant enzymes was evaluated in both filter feeding species. In mussels, a simultaneous induction of SOD/CAT activities was detected between day 1 and 15 in gills and digestive glands, while in clams, the activity of both enzymes was more out-of-phase, with increased SOD between day 1 and 15 in gills, day 1 and 25 in digestive glands, and day 1 and 45 in feet; as for CAT activity, increased SOD was detected between day 1 and 25 in all tissues. Both enzymes play an important role in tissue protection against ROS attack [72]. During this period, both enzymes increased their activity >90% in the different tissues, while in feet, the maximum CAT activity was >40%. This increase in SOD/CAT activities suggests that the initial stage of the bloom involving the assimilation of dinoflagellates and bioaccumulation of toxins leads to the production of superoxide radicals (O_2_^.−^) and hydrogen peroxide (H_2_O_2_) in gills and hepatopancreas [43]. Moreover, in the final phase of the bloom (days 30–70)—which involves the processes of distribution and bioconversion of the toxic analogues—the SOD/CAT enzymes of both species began to decrease their activities until similar levels prior to the bloom were reached. Thus, the increase of SOD and CAT activities may represent an adaptive mechanism against cellular damage preferentially in gills (clams) and digestive glands (mussels and clams) with both enzymes being the defense system against radicals produced by the bloom and toxins [73].

In addition, in the GPx activity, an increase was detected in all the tissues of mussels and clams, with a maximum peak of activity on day 15 in mussels, and on day 25 in clams. This increase in GPx activity may have been a response to the high densities of dinoflagellates, hence to the oxidative conditions to which they had been exposed. However, as the bloom decayed, a decrease in GPx activity was detected (between days 27 and 70), with an increase in the concentrations of toxins preferentially by α-epimers, neoSTX, and STX during the same period, which could lead to high levels of H_2_O_2_, which are inefficiently neutralized [74].

Complementarily, GR activity showed an increase in activity similar to GPx activity in mussels and clams. This increased activity is in alignment with the high levels of the peroxidative component that causes the bloom. However, in the feet of both species, no significant increased activity was detected, which could be due to the fact that it is a tissue whose toxicity is the result of the distribution of biotransformed analogues (GTX1, GTX2, neoSTX and STX); therefore, toxins could cause high oxidative levels in the tissue, leading to an inadequate enzyme level as a response to the high toxicity levels of the detected analogues [75].

Regarding oxidative damage, data showed a significant increase in protein carbonyl levels in all tissues of both bivalves (gills, digestive glands, and feet) with a progressive increase starting on day 25 (50–90%, *p* < 0.05). This increase is directly correlated with the oxidation of proteins (r = 0.957) due to the high production and intensity of reactive oxygen species (ROS) produced during the bloom. On day 70, when the cell density associated with HABs was low (<1−10 cell/L), oxidative damage tended to be an irreversible process, given the high level of damage caused to proteins in visceral and non-visceral tissues of bivalves. These results are complemented by the levels of lipid peroxidation detected in gills, digestive glands, and feet (>50–100%, *p* < 0.05) between day 25 and the end of the bloom. The data suggest that toxins mainly produced by bioconversion (GTX3/GTX2, neoSTX and STX) are distributed throughout the tissues of the species, inducing toxicity and oxidative damage to polyunsaturated fatty acids, which, in species such as mussels and clams, are characterized by a high concentration [76,77], which causes an environment that favors the loss of cell membrane integrity and cell damage due to the formation of protein adducts [47,78].

The loco (*Concholepas concholepas*) is a carnivorous, bottom-dwelling gastropod, whose diet is mainly made up of clams [12,17,79]. Assimilation of STX-group toxins is associated with seafloor browsing during feeding, and with the transfer of toxins through the trophic chain rather than direct interaction with the bloom. The toxicity detected in this species prior to the bloom was ≈86.5 ± 2.4 μg STX equiv 100 g^−1^ with a profile mainly consisting of STX. Previous data have shown that the high biotransformation capacity of this species in its digestive glands allows a rapid conversion of toxic analogues by a reduction pathway, preferably (GTX4/GTX1→neoSTX→STX and GTX3/GTX2→STX) [12]. The variation of the toxic profile is significant between day 15 and day 25, when the presence of GTX3/GTX2 (ratio 1:2) and STX was detected; that ratio was maintained until the end of the analysis period (*p* < 0.05). Enzyme activity over the same period shows a basal level of SOD-CAT that tended to vary only on day 45 (*p* < 0.05) up to day 70 when a higher concentration of toxins was detected in their tissues (≈≈533.7 ± 3.8 μg STX equiv 100 g^−1^). The high levels of activity in both enzymes are directly related to the type of species (predator) and to age, which, in turn, is linked to the size (≈4 years old and ≥15 cm).

High SOD-CAT activity is positively correlated with GPx-GR levels (r = 0.968), which is justifiable due to the high bioconversion capacity of the toxins to be more polar analogues (STX), preferentially in digestive glands, and due to a constant diet characterized by a high prevalence of toxins [80]. In contrast, the oxidative damage detected in feet and digestive glands of locos is high, taking onto consideration the response of the antioxidative defense system. Thus, the damage is correlated with the analogues detected in the tissues of these toxins (r = 0.988), preferentially STX (>90%), which would demonstrate that the high activity of the SOD-CAT and GPx-GR complex is not efficient enough to prevent oxidative damage.

In general, all marine species exposed to xenobiotics induce xenobiotic glutathione-S-transferase (GST) activity, and act in the detoxification process. However, no significant enzyme variation was detected in these species (data not shown), possibly due to the high percentage of bioconversion of the toxins to more water-soluble analogues [81].

Based on the results obtained in this research, we can establish that the variations in antioxidant enzymes and oxidative damage take place through two pathways: (1) in association with a microalgal bloom, and (2) by bioconversion of the toxins making up the STX-group. Regarding the first pathway, Mardones et al. [82] showed a strong relation between the cellular abundance of *Alexandrium catenella* and cytotoxic effects in the rainbow trout cell line RTgill-W1, evidencing that exposure of lysed cells produces more damage in the assimilation phase, which leads to a reduction in cell viability <20%. Thus, the increase of antioxidant enzyme activities in bivalves would be directly related to the interaction and assimilation phase of dinoflagellates (exponential phase of the bloom). Subsequently, during the bioaccumulation process in the digestive glands (>90%), the maximum activity was reached, noting that the lysis of the dinoflagellates would be favored at this stage, which would involve a greater production of ROS, mainly O_2_^.−^ [43,82]. This rapid response of the filter-feeding species could be explained by the exposure to various environmental variables that produce constant episodes of algal blooms, in this case related to *Alexandrium pacificum* [39,83].

**Figure 8:**
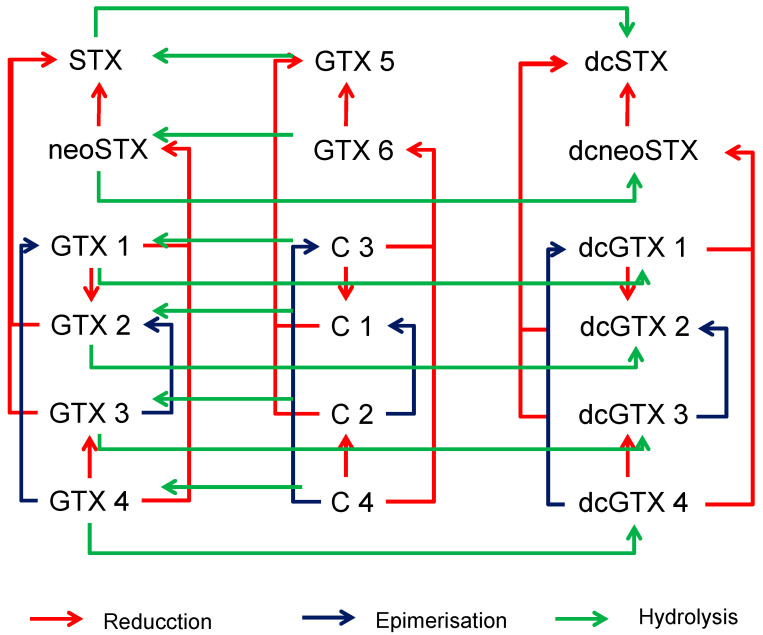
Diagram of the bioconversion pathways detected in molluscs contaminated with STX-group toxins. Adapted from Guéguen et al. [65].

Moreover, the second pathway involving the distribution and bioconversion processes of the toxins from STX-group allows us to establish that the high rate of both processes in visceral tissues (*Mytilis chilensis* and *Ameghinomya antiqua*) and feet (*Concholepas concholepas*) produces high levels of ROS in addition to those produced in the dinoflagellate exposure pathway [84]. STX-group toxins are neurotoxins that induce oxidative stress in marine organisms, so that the high levels of ROS exceed the capacity to be neutralized, thus producing decreased activity of enzyme antioxidants with a consequent increase in oxidative damage in marine species [38,46,85]. Most of the bioconversion processes are mainly carried out in the digestive glands of marine species, and the aim is to increase the polarity of the molecules in order to favor the detoxification of toxins by hydrolysis, epimerization, and reduction reactions (Figure 8).

Finally, climate change may produce an alteration in abiotic and biotic components in different oceanic regions, producing variations in temperature, oxygen concentration, food availability, and alterations in the incidence, prevalence, distribution, and characteristics of HABs [1,8,86,87]. These factors can be important stressor stimuli in aquatic environments due to induction and the imbalance caused by eliminating ROS. [88]. It should be noted that in the larval stages of different species, a low response of antioxidant enzymes has been determined, and therefore, both the bloom and toxins could significantly affect the population of the species.

## 5. Conclusions

In the fjords of southern Chile, the high incidence of *Alexandrium pacificum* leads to increased activity of antioxidant defense enzymes in marine species according to the level of exposure to the bloom with a consequent increase in oxidative damage. Thus, the variability in the oxidative imbalance detected in the different tissues of the species could be related to the processes of microalgal assimilation and cell lysis produced by each species.

## Figures and Tables

**Table 1:** Saxitoxin analogues.

Toxin		R_1_	R_2_	R_3_	R_4_	M.W. ^1^
Saxitoxin	STX	H	H	H	H	299.3
NeoSaxitoxin	neoSTX	OH	H	H	H	315.3
Gonyautoxin-1	GTX-1	OH	H	OSO_3_^−^	H	411.4
Gonyautoxin-2	GTX-2	H	H	OSO_3_^−^	H	395.4
Gonyautoxin-3	GTX-3	H	OSO_3_^−^	H	H	395.4
Gonyautoxin-4	GTX-4	OH	OSO_3_^−^	H	H	411.4
Gonyautoxin-5	GTX-5	H	H	H	SO_3_^−^	379.4
Gonyautoxin-6	GTX-6	OH	H	H	SO_3_^−^	395.4
N-21 Sulfocarbamoyl-GTX2	C1	H	H	OSO_3_^−^	SO_3_^−^	475.4
N-21 Sulfocarbamoyl-GTX3	C2	H	OSO_3_^−^	H	SO_3_^−^	475.4
N-21 Sulfocarbamoyl-GTX1	C3	OH	H	OSO_3_^−^	SO_3_^−^	491.4
N-21 Sulfocarbamoyl-GTX4	C4	OH	OSO_3_^−^	H	SO_3_^−^	491.4

^1^ Molecular weight (g/mol).

## Data Availability

The authors confirm that the original/raw data supporting the findings of this study are available within the article.
